# Seasonal Variation of Dystocia in a Large Danish Cohort

**DOI:** 10.1371/journal.pone.0094432

**Published:** 2014-04-15

**Authors:** Christine Rohr Thomsen, Niels Uldbjerg, Lone Hvidman, Hjördís Ósk Atladóttir, Tine Brink Henriksen, Ioanna Milidou

**Affiliations:** 1 Department of Obstetrics and Gynecology, Aarhus University Hospital, Skejby, Aarhus, Denmark; 2 Department of Pediatrics, Aarhus University Hospital, Skejby, Aarhus, Denmark; 3 Perinatal Epidemiology Research Unit, Institute of Clinical Medicine, Aarhus University Hospital, Skejby, Aarhus, Denmark; Iran University of Medical Sciences, Iran (Islamic Republic Of)

## Abstract

**Background:**

Dystocia is one of the most frequent causes of cesarean delivery in nulliparous women. Despite this, its causes are largely unknown. Vitamin D receptor (VDR) has been found in the myometrium. Thus, it is possible that vitamin D affects the contractility of the myometrium and may be involved in the pathogenesis of dystocia. Seasonal variation of dystocia in areas with distinct seasonal variation in sunlight exposure, like Denmark, could imply that vitamin D may play a role. This study examined whether there was seasonal variation in the incidence of dystocia in a Danish population.

**Method:**

We used information from a cohort of 34,261 nulliparous women with singleton pregnancies, spontaneous onset of labor between 37 and 42 completed gestational weeks, and vertex fetal presentation. All women gave birth between 1992 and 2010 at the Department of Obstetrics and Gynecology, Aarhus University Hospital, Skejby. Logistic regression combined with cubic spline was used to estimate the seasonal variation for each outcome after adjusting for calendar time.

**Results:**

No evidence for seasonal variation was found for any of the outcomes: acute cesarean delivery due to dystocia (p = 0.44); instrumental vaginal delivery due to dystocia (p = 0.69); oxytocin augmentation due to dystocia (p = 0.46); and overall dystocia (p = 0.91).

**Conclusion:**

No seasonal variation in the incidence of dystocia was observed in a large cohort of Danish women. This may reflect no association between vitamin D and dystocia, or alternatively that other factors with seasonal variation and influence on the occurrence of dystocia attenuate such an association.

## Introduction

Dystocia is defined as slow, abnormal progression of labor [1], [2], [3]. It is the result of problems related to either the passenger, such as abnormal fetal presentation; the passage, such as absolute or relative cephalopelvic disproportion; or the power, such as insufficient uterine activity [1].Known risk factors for dystocia include nulliparity, labor induction, high maternal age, short stature [1], [4], [5], postterm gestation, high birth weight and abnormal vertex presentation. [4], [5]. It is also debated whether epidural analgesia itself may cause dystocia [4], [5], [6], [7], [8]. Sub-fecundity, which has increased due to the higher age of nulliparous women, may also play a role, as failure to progress is frequently observed in pregnancies achieved by assisted reproductive technology. [5], [9]. Despite the liberal use of oxytocin augmentation, pregnancies complicated by dystocia often end with assisted deliveries, including forceps, vacuum or, commonly, caesarean section. It is estimated that dystocia accounts directly or indirectly for up to 30–60% of all caesarean deliveries in the United States [3], [10], [11].

Recent studies associated levels of vitamin D in pregnancy with complications such as gestational diabetes, preeclampsia, bacterial vaginosis, and preterm birth. They attempted to define the optimal levels of vitamin D in serum during pregnancy, and to recommend the optimal dose for supplementation [12], [13], [14].The prevalence of vitamin D deficiency during pregnancy varies from 18% to 84% [15], [16], [17] depending on the geographic area of residency and local clothing customs. An established symptom of vitamin D deficiency is proximal skeletal muscle weakness, caused by reduced stimulation of the cellular Vitamin D Receptor (VDR) [18], [19]. Several studies have described VDR expression in non-vascular smooth muscle [19] including the myometrium [20]. Thus, it is possible that vitamin D affects the contractility of the myometrium and may be involved in the risk of dystocia. In addition, calcium levels are affected by the concentration of vitamin D. Calcium itself also seems to play a part in myometrial contractility, by affecting the production of prostaglandins and the action of nitric oxidase [21]. Low concentration of extracellular calcium or inhibition of the entry of the ion into the myometrium cell reduces the contractility of the muscle fibers and their sensitivity to oxytocin [22].

If vitamin D deficiency causes dystocia, seasonal variation in the incidence of dystocia would be expected, particularly in areas with distinct seasonal variation in sunlight exposure. In northern latitude regions, such as Denmark, ultraviolet B (UVB) radiation is nonexistent from October until April [23]. The resulting lack of dermal vitamin D production during this period is reflected in a seasonal variation in plasma 25-hydroxyvitamin D (P-25OHD) [24], causing up to 90% of Danish women to be vitamin D deficient (<50 nmol/l) during winter. By contrast, this number is reduced to under one-fifth during summer [25]. Seasonal variation in the incidence of dystocia, if found, would necessitate further studies on actual levels of vitamin D and dystocia. If vitamin D depletion increases the risk of dystocia, it would imply that an inexpensive, safe, and easily accessible method of preventing dystocia and associated complications is readily available. This study therefore examined whether there is seasonal variation in dystocia in a Danish cohort of pregnant women.

## Materials and Methods

### Study population

Our study was based on data from the Aarhus Birth Cohort (ABC), which includes all women who have given birth at the Department of Obstetrics and Gynecology, Aarhus University Hospital, Denmark, since 1989 [26].

The attending midwife registers information on labor induction and course of birth immediately after delivery using structured birth registration forms. Before data entry, the registration forms are manually checked for inconsistency and cross-checked with the medical record by a trained research midwife. Information about general health status, lifestyle, ethnicity, educational level and obstetric and medical history from all pregnant women living permanently in the area is routinely collected by a self-administered questionnaire completed around 12 weeks of gestation, i.e. before delivery.

We included nulliparous women who gave birth to singletons at term (between 37 and 41 completed gestational weeks) between 1992 and 2010, with spontaneous onset of labor by either contractions or rupture of membranes, and intended vaginal delivery (n = 35,589). We excluded women with fetus in non-vertex cephalic (n = 1,208) and in breech (n = 64) presentation, intrauterine fetal death (n = 5), and women with incomplete registration of the outcome variables (n = 51), leading to a study population of 34,261 women. Background data from the questionnaires were available for 29,465 (86%) of these women.

The study was approved by the Danish Data Protection Agency (journal number: 2011-41-6652) and the Danish National Board of Health (journal number: 3-3013-36/1/KWH). Data from clinical records can be used without informed consent in studies approved by the Danish National Board of Health if they are treated anonymously, as was the case in this study. It is possible to access The Danish Data Archive, where all data used in this study is deposited [27].

### Statistical analysis

All analysis was performed with STATA version 11.0 (StataCorp, College Station, TX).

In order to estimate seasonal variation, the effect of changes in the incidence of the outcomes over time, if any, needed to be removed. This is because an increase in incidence over the study period can impose an artifact of higher incidence of dystocia in December than January, as months pool through the years. Such artifacts can be falsely interpreted as seasonal variation [28]. We investigated the presence of such time trends by data inspection and tabulation, without using trend analyses techniques. We therefore adjusted for time trend, i.e., the calendar time from 1 January 1992 until 31 December 2010, using three spline variables. This analytical approach offers a relatively high degree of freedom with few parameters.

We estimated the risk of dystocia with possible seasonal variation by time of birth separately for each outcome after adjusting for time trend using the cubic spline method [29]. The cubic spline model with seasonality in a binomial regression model seems to be the simplest and most robust analytic method that can adjust for increasing incidence of the outcome and at the same time study seasonality in a binary outcome [30]. The cubic spline is a smoothing technique that consists of piecewise cubic polynomials as a set of explanatory variables (the spline variables) in a regression analysis. We performed our analysis under the assumption of a periodic season, which involved two restrictions: 1. The risk estimates at the end of each December were restricted to be equal to the risk estimates at the beginning of January; and 2. The slope of the curve at the end of the year was restricted to be equal to the slope of the curve at the beginning of the year. We fitted a model with three equally spaced knots (six spline variables reduced to four due to the periodic season assumption).

The final model logistic regression was performed for each outcome separately, using seven spline variables (four for the seasonal variation and three for the adjustment for time trend). The hypothesis of seasonal variation of dystocia was tested against the null hypothesis of no seasonal variation by testing significance of all four seasonal spline variables simultaneously. Statistical significance was defined as a two-tailed p value of less than 0.05.

The cubic spline method for studying the incidence of dystocia by time of delivery offers the advantage of a day-by-day analysis of seasonal variation, thus avoiding grouping the year into months or other longer time intervals. The resulting relative risk (RR) curves compare the risk of dystocia on each day of delivery to the average risk of the entire year. The 95% confidence interval (CI) was calculated according to the fit of the spline model, i.e., the confidence interval corresponds to the fit evaluated for each individual day. In order to demonstrate the adequacy of the seasonal variation obtained by the cubic spline function, we estimated relative risk by grouping the season into months of delivery, still adjusting for time trend as described above.

### Outcome

In order to achieve higher specificity of the outcome, only women with an intervention due to dystocia were classified as having dystocia. We studied seasonal variation in four groups of women:

1. Women who delivered by emergency cesarean delivery indicated by dystocia, where emergency cesarean delivery is defined as a cesarean delivery carried out within eight hours of the decision.
2. Women who delivered by vacuum or forceps (instrumental deliveries) indicated by dystocia.
3. Women who received oxytocin augmentation due to dystocia at a spontaneous cervical dilation of 4 cm or more.
4. Women with any of the above (dystocia).

Only one type of delivery was registered per woman, thus Groups 1 and 2 are mutually exclusive. However, a relatively small number of women who received a trial of labor augmentation by oxytocin followed by an acute cesarean delivery or an instrumental delivery are represented in both Groups 1 and 3 (n = 472), or 2 and 3 (n = 1,269). Women who delivered by cesarean delivery indicated by a preceding failed attempt of instrumental vaginal delivery are included in Group 1, but only in cases where dystocia was also stated as the indication for the cesarean delivery.

Dystocia may be related to factors acting at different times during pregnancy; both at the time the myometrium grows and develops, and after it has reached its final size. If such factors change during the year, and the length of gestation is not considered, then seasonal variation in pregnancy exposures arising at times other than close to delivery may be missed. We therefore decided to restrict our analysis to infants born at term (gestational age from 37^+0^ to 41^+6^). In order to eliminate the effect of variations in gestational length for pregnancies carried at full term, we further investigated seasonal variation of dystocia according to time of conception, calculated by subtraction of the gestational age minus 14 days from the date of delivery.

In our primary analysis, we did not adjust for risk factors for dystocia or vitamin D deficiency, because they were considered unlikely to show any seasonal variation. In order to confirm this, we examined the proportion of infants weighing more than 4,000 grams at birth (from birth registration, data available for all births) and of infants born to smoking mothers (in four groups), mothers in five age groups, mothers in four pre-pregnancy body mass index (BMI) groups, mothers in four ethnicity groups, and mothers in four educational level groups (from self-administered questionnaire, data available for 86% women) by month of birth. As expected, none of these risk factors showed any seasonal variation, and they were not included in the primary analyses. However, birth weight, maternal pre-pregnancy BMI and maternal ethnicity, factors associated with dystocia and with the metabolism of vitamin D, showed some fluctuation during the study period that could create an artifact of seasonal variation despite adjustment for calendar time. In secondary analyses, we estimated the RR for dystocia for each month of delivery adjusted for maternal pre-pregnancy BMI (missing n = 6,000), for birth weight (missing n = 27), for ethnicity (missing n = 10.353) and for educational level (missing n = 13.039).

We studied the seasonal variation of cesarean and instrumental delivery regardless of indication, as well as all women registered as having dystocia irrespective of interventions due to dystocia. Furthermore, we studied whether changing the parameter regarding cervical dilation from 4 cm to 6 cm for initiating oxytocin augmentation would change the result [31]. Finally, we performed three different analyses, grouping the year into four quarters, testing whether using the day-by-day analysis outperforms potentially smaller associations. The first analysis was performed by splitting the year into the quarters of Jan-March, April-Jun, Jul-Sep and Oct-Dec. Hereafter, we ran two additional analyses shifting the quarters by one month.

## Results

Of the 34,261 women included in the study, 751 (2.2%) delivered by acute cesarean section because of dystocia, 1,711 (5%) delivered by vacuum or forceps because of dystocia, and 9,929 (29%) received oxytocin augmentation due to dystocia after spontaneous dilatation of the orificium to 4 cm or more. Overall dystocia was observed in 10,655 women (31.1%); the remaining 23,606 women comprised the reference group.

All four outcomes showed non-monotonous time trends. The tendency over time was towards more cesarean deliveries (from 0.4% to 3.7%), fewer instrumental deliveries (from 8% to 2%) and more oxytocin augmentations (from 29% to 66%). Over time, a greater number of deliveries seemed to be characterized by dystocia (from 25% to 36%). During the time of the study, changes in diagnostic criteria and in criteria for intervention occurred. This may partially explain the observed time trends.

The background characteristics of women in each group are shown in Table 1. Women who received an intervention due to dystocia tended to have higher levels of education, to be older, to be of shorter stature, to have higher pre-pregnancy BMI, and to give birth to larger infants. This was most pronounced for women who delivered by emergency caesarian section due to dystocia.

**Table 1:** Background characteristics of nulliparous women with dystocia, the Aarhus Birth Cohort, Denmark (1992–2010).

		Reference group (n = 23,606)	Acute cesarean because of dystocia (n = 751)	Instrumental delivery because of dystocia (n = 1,711)	Oxytocin augmentation because of dystocia (n = 9,929)	Dystocia (n = 10,655)
Cigarette smoking (cigarettes per day)*						
	Nonsmoker	12,997 (85.2%)	521 (88.6%)	1,251 (86.7%)	6,807 (86.9%)	7,301 (86.9%)
	1–4	540 (3.5%)	17 (2.9%)	44 (3.1%)	261 (3.3%)	277 (3.3%)
	5–9	753 (4.9%)	25 (4.3%)	78 (5.4%)	359 (4.6%)	396 (4.7%)
	10+	962 (6.3%)	25 (4.3%)	70 (4.9%)	403 (5.2%)	429 (5.1%)
Alcohol intake (units# per week)*						
	<1	11,720 (81.8%)	475 (87.3%)	1,150 (82.0%)	6,131 (82.5%)	6,609 (82.7%)
	1–2	2,148 (15.0%)	53 (9.7%)	208 (14.8%)	1,066 (14.4%)	1,136 (14.2%)
	3+	454 (3.2%)	16 (2.9%)	45 (3.2%)	233 (3.1%)	245 (3.1%)
Education*						
	Unemployed, unskilled	3,494 (25.7%)	122 (21.9%)	284 (21.4%)	1,609 (22.6%)	1,737 (22.7%)
	Skilled, students	2,033 (15.0%)	70 (12.6%)	206 (15.6%)	1,018 (14.3%)	1,100 (14.4%)
	Further education (FE) 1–2year	1,960 (14.4%)	75 (13.5%)	200 (15.1%)	1,072 (15.1%)	1,138 (14.9%)
	FE 3+ year	6,096 (44.9%)	290 (52.1%)	635 (47.9%)	3,413 (48.0%)	3,664 (48.0%)
Maternal pre-pregnancy weight (kg)*						
	<50	818 (4.3%)	29 (4.1%)	77 (5.0%)	424 (4.8%)	453 (4.7%)
	50–59	6,137 (32.5%)	204 (29.0%)	549 (35.6%)	2,888 (32.5%)	3,113 (32.6%)
	60–69	7,326 (38.8%)	246 (35.0%)	568 (36.8%)	3,412 (38.4%)	3,631 (38.0%)
	70–79	3,003 (15.9%)	124 (17.6%)	237 (15.4%)	1,391 (15.6%)	1,502 (15.7%)
	80+	1,599 (8.5%)	100 (14.2%)	113 (7.3%)	783 (8.8%)	855 (9.0%)
Maternal height (cm)*						
	<160	1,412 (7.5%)	119 (16.9%)	143 (9.2%)	822 (9.2%)	897 (9.4%)
	160–169	9,108 (48.1%)	392 (55.5%)	813 (52.5%)	4,503 (50.4%)	4.874 (50.8%)
	170–179	7,589 (40.1%)	180 (25.5%)	544 (35.1%)	3,301 (37.0%)	3,500 (36.5%)
	180+	819 (4.3%)	15 (2.1%)	50 (3.2%)	304 (3.4%)	325 (3.4%)
Maternal pre-pregnancy BMI*						
	<18.5	1,143 (6.1%)	23 (3.3%)	84 (5.5%)	491 (5.6%)	515 (5.4%)
	18.5–24	14,149 (75.8%)	454 (64.8%)	1,180 (77.3%)	6,565 (74.4%)	7,037 (74.3%)
	25–29	2,539 (13.6%)	154 (22.0%)	197 (12.9%)	1,315 (14.9%)	1,426 (15.1%)
	30+	839 (4.5%)	70 (10.0%)	66 (4.3%)	448 (5.1%)	496 (5.2%)
Maternal age (years)*						
	<20	637 (3.3%)	8 (1.3%)	19 (1.3%)	206 (2.5%)	214 (2.4%)
	20–24	2,937 (15.0%)	44 (7.1%)	153 (10.7%)	976 (12.0%)	1,034 (11.8%)
	25–29	7,973 (40.7%)	249 (40.0%)	618 (43.3%)	3,469 (42.7%)	3,716 (42.4%)
	30–35	5,632 (28.7%)	213 (34.2%)	455 (31.8%)	2,514 (30.9%)	2,725 (31.1%)
	35+	2,437 (12.4%)	108 (17.4%)	184 (12.9%)	969 (11.9%)	1,072 (12.4%)
Birth weight (g)*						
	>4000	2,628 (11.2%)	270 (36.0%)	380 (22.2%)	1,872 (18.9%)	2,044 (19.2%)
	≤4000	20,948 (88.9%)	481 (64.1%)	1,329 (77.7%)	8,051 (81.1%)	8,605 (80.8%)
Ethnicity*						
	Danes/European/Australians	13,848 (89.8%)	523 (88.5%)	1,333 (91.5%)	7,167 (90.6%)	7,686 (90.6%)
	Middle East/Northern Africans	866 (5.6%)	26 (4.4%)	65 (4.5%)	404 (5.1%)	435 (5.1%)
	Asian/Greenlanders	403 (2.6%)	30 (5.1%)	49 (3.4%)	222 (2.8%)	244 (2.9%)
	Africans/South-central Americans	303 (2.0%)	12 (2.0%)	10 (0.7%)	116 (1.5%)	123 (1.5%)

Note that the four groups are not mutually exclusive.

*Missing up to 42.5% because of women who did not return the self-administered questionnaire.

# One unit of alcohol corresponds to 12 gram of alcohol.

**Figure 1:**
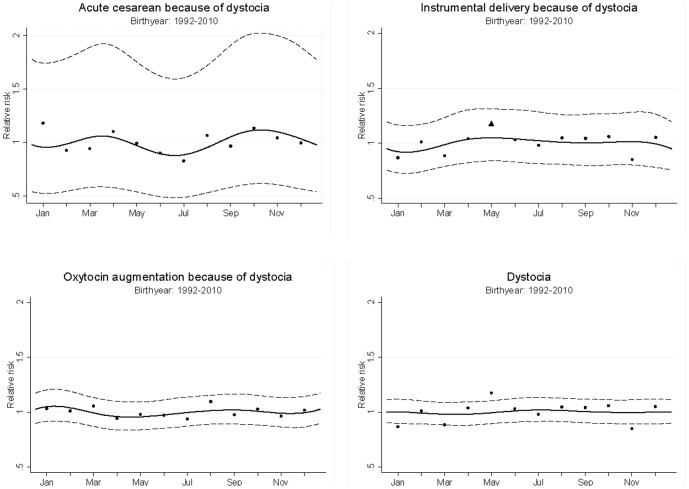
Seasonal variation of dystocia. The solid line represents the estimated seasonal variation of dystocia after adjusting for time-trend, with 95% CI (dashed line). Dots represent the relative risk (RR) obtained by grouping the season into months. Some monthly point estimates (dots) are outside the CIs of the seasonal variation curve, and one is significant, (marked with a triangle).

Figure 1 shows the seasonal variation in emergency cesarean delivery, instrumental vaginal delivery, oxytocin augmentation due to dystocia, and overall dystocia according to date of delivery. The figure presents the seasonal variation from the spline method, with 95% confidence intervals. The monthly relative risk estimates without smoothing technique, but after adjustment for time trend, can also be seen in Figure 1. The relative risk estimates (RR) for the four outcomes at different periods during the year (with 95% confidence intervals shown in brackets) varied from 0.7 [0.48–1.00] to 1.35 [1.06–1.72]. The women who gave birth by instrumental vaginal delivery because of dystocia had the largest variation in relative risk estimates, ranging from the lowest risk estimate of 0.94 [0.72–1.22] for women giving birth in November to the highest risk estimates of 1.35 [1.06–1.72] for women giving birth in May. Dystocia overall had the smallest variation in risk estimates, ranging from 0.95 [0.84–1.06] for women giving birth in November to 1.09 [0.98–1.22] for women giving birth in August. The less frequent outcomes, such as emergency cesarean delivery because of dystocia, showed a greater variation in risk estimates during the year, while the more frequent outcomes, such as dystocia overall, presented minor variation in risk estimates during the year.

No evidence for seasonal variation was found for any of the outcomes: emergency cesarean delivery due to dystocia (p = 0.44); instrumental vaginal delivery due to dystocia (p = 0.69); oxytocin augmentation due to dystocia (p = 0.46); and overall dystocia (p = 0.91). Similar results were yielded when we investigated seasonal variation in the incidence of dystocia according to date of conception (results not shown).

There were no changes in these results based on the secondary analysis, in which the estimated relative risk for dystocia for each month of delivery was adjusted for pre-pregnancy BMI, birth weight of the child, maternal ethnicity or educational level (results not shown). Moreover, we found no seasonal variation in the overall incidence of emergency cesarean delivery (p = 0.9) regardless of indication, nor of overall instrumental vaginal delivery (p = 0.7), overall emergency cesarean delivery and instrumental vaginal delivery together (p = 0.6), or women registered as having dystocia irrespective of interventions due to dystocia (p = 0.60). Furthermore, we found no seasonal variation in the incidence of oxytocin augmentation due to dystocia after changing the parameter regarding cervical dilation from 4 to 6 cm (p = 0.69). Finally, grouping the year into quarters did not change the result.

## Discussion

Our results show no seasonal variation in the incidence of dystocia, defined as emergency cesarean delivery, instrumental vaginal delivery, or oxytocin augmentation all indicated by dystocia, as well as overall dystocia, in a Danish cohort. Some tendency towards more dystocia-indicated cesarean deliveries during the spring and fall was observed, but the results were not statistically significant.

To our knowledge, no previous study has tested the association between season of birth and the risk of dystocia. Two previous studies examined the association between serum vitamin D and acute cesarean delivery, but they showed conflicting results. The first study demonstrated no association between a low level (<30 nmol/l) of vitamin D and the risk of an acute cesarean delivery in a Pakistani population [32]. However, the women included in that study were characterized by malnourishment in addition to their vitamin D deficiency; moreover, the clothing customarily worn by Pakistani women may predispose them to vitamin D deficiency. The second study, from Boston, demonstrated that nulliparous women who delivered by cesarean section had lower serum vitamin D compared to controls who delivered vaginally (P-25OHD median of 45 nmol/liter and 63 nmol/liter, respectively). However, in this study dystocia was the indication for cesarean delivery in only 17 of the 43 cases, while the rest had a cesarean delivery indicated by non-reassuring fetal tracing, fetal malpresentation, or other indications [33]. Both studies have a number of limitations, which were addressed in the present study. Unlike previous studies, we used the spline technique with a day-by-day analysis and tested for seasonal variation using four degrees of freedom after adjusting for time trends. Furthermore, this study involved a large cohort of healthy women who were relatively homogeneous in terms of socioeconomic and nutritional status. All background information used for the secondary analyses was collected by self-administered questionnaires at gestational week 12, and is therefore less prone to recall biases. Generally, dystocia is difficult to define by objective measures and to date there is no consensus for the diagnostic criteria [34]. This lack of specificity in the outcome definition, which is unrelated to the season of birth, would lead to non-differential misclassification and thus cause bias towards no association. We tried to increase the validity of the outcome assessment by using well-registered interventions as proxies, and still no significant seasonal variation was found. Nonetheless it is possible that the lack of seasonal variation in our study is partially explained by some misclassification of dystocia, especially in the oxytocin augmentation group.

According to our primary hypothesis, plasma 25OHD variations due to variation in exposure to sunlight according to the date of delivery should cause a seasonal variation in the risk of dystocia. However, plasma 25OHD concentrations among women may vary as a consequence of differences in eating habits as well as sun exposure, and the fact that we did not directly measure plasma 25OHD is a limitation of the present study. Additionally, plasma 25OHD concentration at other gestational ages may also be important for delivery, e.g. at the time of conception and early in pregnancy, where the myometrium starts to grow and develop. Such potential vitamin D-mediated effects may counteract each other and lead to negative results, even though we restricted our study to term deliveries in order to reduce some of the variation in vitamin D levels in earlier stages of pregnancy and repeated the analysis according to conception dates. Furthermore, restricting our study to term deliveries excludes women with preeclampsia and preterm deliveries, both associated with vitamin D deficiency. This may have caused selection of women with less variation in their plasma 25OHD, reducing the ability to find an association with dystocia.

Seasonal variation according to month of birth has been seen in some immune system-related diseases of pregnancy, such as preeclampsia [35], [36]. The immune system is not known to be involved in the pathogenesis of dystocia. However, if any other factors subject to seasonal change, such as eating habits or exposure to viral infections, or meteorological factors, such as humidity, temperature or exposure to daylight [28], affect myometrial function, we would expect to see a seasonal variation in dystocia. With the present study design, it could be difficult to isolate the association between vitamin D and dystocia. If these factors pull in different directions, the net association without adjustment for those in our study would be no association. Finally, dystocia is not only caused by insufficient contractility of the myometrium, but it is also affected by the size and position of the fetus, the pelvic conditions, and the cervical collagen concentration [37]. In our study we included infants with vertex presentation and adjusted in a secondary analysis for birth weight, but, clearly, risk factors for dystocia that were not taken into account could cause bias towards no association. Our results therefore do not preclude the possibility that vitamin D is of importance in the pathogenesis of dystocia, but indicate the need for further studies with strict definition of dystocia, measurements of actual levels of vitamin D in different stages of pregnancy, and exclusion of causes of dystocia other than myometrial function.

The external validity of the study should most likely be restricted to geographical areas with sunlight exposure, dietary and clothing habits similar to those in Denmark. The Danish National Board of Health recently increased the amount of recommended daily vitamin D intake for pregnant women to 10 µg of supplement during the entire pregnancy, but the exact amount of vitamin D supplementation during pregnancy that is necessary in order to achieve a beneficial effect remains unknown. Our study did not supply evidence for an association between vitamin D and dystocia, but more direct measures of vitamin D status in pregnancy should be studied in relation to pregnancy and delivery complications in order to draw more certain conclusions.
